# 2-(2,2-Dimethyl-2,3-dihydro-1-benzofuran-7-yl­oxy)acetic acid monohydrate

**DOI:** 10.1107/S1600536810018659

**Published:** 2010-05-22

**Authors:** Lin-Tao Yang, Jiao Ye, Xian-Fu Luo, Ai-Xi Hu

**Affiliations:** a College of Chemistry and Chemical Engineering, Hunan University, Changsha 410082, People’s Republic of China

## Abstract

In the title compound, C_12_H_14_O_4_·H_2_O, the dihydro­benzo­furan ring adopts an envelope conformation with the substituted C atom 0.142 (1) Å out of the least-squares plane. In the crystal, the components are linked *via* inter­molecular O_water_—H⋯O and O—H⋯O_water_ hydrogen-bonding inter­actions, forming a three-dimensional network.

## Related literature

For background to carbamate-based insecticides, see: Xu *et al.* (2005 ▶); Li *et al.* (2009 ▶).
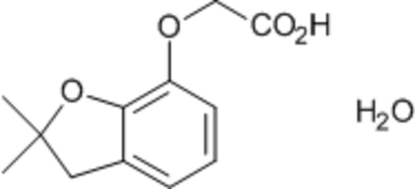


## Experimental

### 

#### Crystal data

C_12_H_14_O_4_·H_2_O
*M* *_r_* = 240.25
Monoclinic, 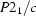

*a* = 10.1692 (7) Å
*b* = 9.2516 (6) Å
*c* = 15.3647 (11) Å
β = 121.000 (1)°
*V* = 1239.06 (15) Å^3^
*Z* = 4
Mo *K*α radiation
μ = 0.10 mm^−1^
*T* = 173 K
0.46 × 0.42 × 0.30 mm

#### Data collection

Bruker SMART 1000 CCD diffractometer
Absorption correction: multi-scan (*SADABS*; Sheldrick, 2004 ▶) *T*_min_ = 0.955, *T*_max_ = 0.971
6151 measured reflections
2697 independent reflections
2120 reflections with *I* > 2σ(*I*)
*R*_int_ = 0.019

#### Refinement

*R*[*F* ^2^ > 2σ(*F* ^2^)] = 0.039
*wR*(*F* ^2^) = 0.111
*S* = 1.04
2697 reflections
163 parameters
H atoms treated by a mixture of independent and constrained refinement
Δρ_max_ = 0.25 e Å^−3^
Δρ_min_ = −0.16 e Å^−3^

### 

Data collection: *SMART* (Bruker, 2001 ▶); cell refinement: *SAINT-Plus* (Bruker, 2003 ▶); data reduction: *SAINT-Plus*; program(s) used to solve structure: *SHELXTL* (Sheldrick, 2008 ▶); program(s) used to refine structure: *SHELXTL*; molecular graphics: *SHELXTL*; software used to prepare material for publication: *SHELXTL*.

## Supplementary Material

Crystal structure: contains datablocks I, global. DOI: 10.1107/S1600536810018659/wm2329sup1.cif

Structure factors: contains datablocks I. DOI: 10.1107/S1600536810018659/wm2329Isup2.hkl

Additional supplementary materials:  crystallographic information; 3D view; checkCIF report

## Figures and Tables

**Table 1:** Hydrogen-bond geometry (Å, °)

*D*—H⋯*A*	*D*—H	H⋯*A*	*D*⋯*A*	*D*—H⋯*A*
O5*W*—H5*A*⋯O1	0.86 (2)	1.95 (2)	2.8104 (15)	173.0 (19)
O5*W*—H5*B*⋯O3	0.85 (2)	1.94 (2)	2.7888 (15)	176.5 (19)
O4—H4*A*⋯O5*W*^i^	0.84	1.71	2.5416 (15)	171

Symmetry code: (i) 
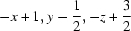
.

**Fig. 1.**
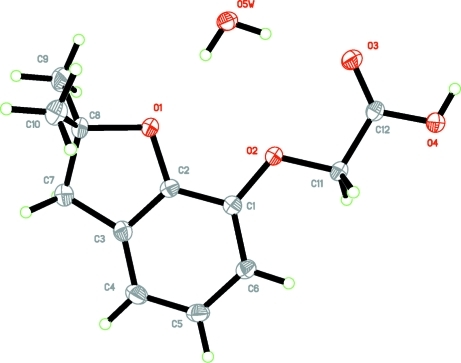
The molecular structure of the title compound showing 30% probability displacement ellipsoids. H atoms are drawn as spheres with arbitrary radius.

**Fig. 2.**
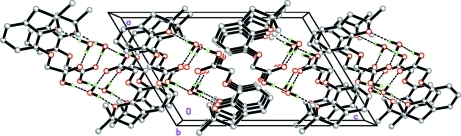
A packing diagram for the title compound, viewed down [010]. H atoms bonded to C atoms have been omitted for clarity.

## supplementary crystallographic information

### Comment

2-(2,2-dimethyl-2,3-dihydrobenzofuran-7-yloxy)acetic acid monohydrate (I), C_12_H_14_O_4_^.^H_2_O, is a derivative of commercially available Carbofuran which is a carbamate-based insecticide (Xu *et al.*, 2005; Li,*et al.*, 2009), Herein we report the synthesis and structure of the title compound.

The dihedral angle between the plane C7—C3—C2—O1 and the plane C8—O1—C7 is 23.20 (14)°, which indicates that the dihydrobenzofuran ring is in an envelope conformation (Fig. 1). Its substituted C8 atom is 0.142 (1) Å out of the least-squares plane defined by O1, C2, C3, C7 and C8. In the crystal structure, intermolecular O_water_—H···O and and O—H···O_water_ hydrogen bonds link organic molecules and water molecules into a three-dimensional network (Fig. 2).

### Experimental

0.10 mol 2,2-dimethyl-2,3-dihydrobenzofuran-7-ol , 0.12 mol chloroacetic acid, 0.25 mol sodium hydroxide and 70 ml water were stirred and heated under reflux for 3 h. Then the reaction mixture was cooled to 283 K and 15 ml concentrated hydrochloric acid was added to give 2-(2,2-dimethyl-2,3-dihydrobenzofuran-7-yloxy)acetic acid hydrate as amber solid of 21.91 g, yield 98.5%. Single colourless crystals suitable for X-ray diffraction were obtained by slow evaporation of an ethyl acetate solution at room temperature over a period of nine days.

^1^H NMR (CDCl_3_,300 MHz), δ: 1.50(s, 6H, 2CH_3_), 3.03(s,2H,CH_2_), 4.71(s,2H,OCH_2_), 6.74~6.83(m, 2H,ArH),  6.84~6.87(m,1H,ArH).

### Refinement

All H atoms except for the water H atoms were refined in the riding-model approximation, with C—H distances of 0.98 Å (methyl), 0.95Å (aromatic) and 0.99Å (methylene), and with *U*iso(H)=1.5 or 1.2*U*eq(carrier). The water H atoms were located in Fourier syntheses. Their positions were refined with distance restraints of 0.85 (2) Å, with *U*iso(H) values set equal to 1.5*U*eq(O). The carboxylate proton was placed in a calculated position.

### Crystal data

**Table:**

C_12_H_14_O_4_·H_2_O	*F*(000) = 512
*M**_r_* = 240.25	*D*_x_ = 1.288 Mg m^−^^3^
Monoclinic, *P*2_1_/*c*	Melting point: 383.2 K
Hall symbol: -P 2ybc	Mo *K*α radiation, λ = 0.71073 Å
*a* = 10.1692 (7) Å	Cell parameters from 3163 reflections
*b* = 9.2516 (6) Å	θ = 2.3–27.1°
*c* = 15.3647 (11) Å	µ = 0.10 mm^−^^1^
β = 121.000 (1)°	*T* = 173 K
*V* = 1239.06 (15) Å^3^	Block, colorless
*Z* = 4	0.46 × 0.42 × 0.30 mm

### Data collection

**Table:**

Bruker SMART 1000 CCD diffractometer	2697 independent reflections
Radiation source: fine-focus sealed tube	2120 reflections with *I* > 2σ(*I*)
graphite	*R*_int_ = 0.019
ω scans	θ_max_ = 27.1°, θ_min_ = 2.3°
Absorption correction: multi-scan (*SADABS*; Sheldrick, 2004)	*h* = −12→11
*T*_min_ = 0.955, *T*_max_ = 0.971	*k* = −7→11
6151 measured reflections	*l* = −19→19

### Refinement

**Table:**

Refinement on *F*^2^	Primary atom site location: structure-invariant direct methods
Least-squares matrix: full	Secondary atom site location: difference Fourier map
*R*[*F*^2^ > 2σ(*F*^2^)] = 0.039	Hydrogen site location: inferred from neighbouring sites
*wR*(*F*^2^) = 0.111	H atoms treated by a mixture of independent and constrained refinement
*S* = 1.04	*w* = 1/[σ^2^(*F*_o_^2^) + (0.0517*P*)^2^ + 0.395*P*] where *P* = (*F*_o_^2^ + 2*F*_c_^2^)/3
2697 reflections	(Δ/σ)_max_ = 0.001
163 parameters	Δρ_max_ = 0.25 e Å^−^^3^
0 restraints	Δρ_min_ = −0.16 e Å^−^^3^

### Special details

**Table:**

Experimental. ^1^H NMR (CDCl_3_,300 MHz), delta: 1.50(s, 6H, 2CH_3_), 3.03(s,2H,CH_2_), 4.71(s,2H,OCH_2_), 6.74~6.83(m, 2H,ArH), 6.84~6.87(m,1H,ArH).
Geometry. All esds (except the esd in the dihedral angle between two l.s. planes) are estimated using the full covariance matrix. The cell esds are taken into account individually in the estimation of esds in distances, angles and torsion angles; correlations between esds in cell parameters are only used when they are defined by crystal symmetry. An approximate (isotropic) treatment of cell esds is used for estimating esds involving l.s. planes.
Refinement. Refinement of *F*^2^ against ALL reflections. The weighted *R*-factor wR and goodness of fit *S* are based on *F*^2^, conventional *R*-factors *R* are based on *F*, with *F* set to zero for negative *F*^2^. The threshold expression of *F*^2^ > σ(*F*^2^) is used only for calculating *R*-factors(gt) etc. and is not relevant to the choice of reflections for refinement. *R*-factors based on *F*^2^ are statistically about twice as large as those based on *F*, and *R*- factors based on ALL data will be even larger.

### Fractional atomic coordinates and isotropic or equivalent isotropic displacement parameters (Å^2^)

**Table:**

	*x*	*y*	*z*	*U*_iso_*/*U*_eq_	
C1	0.31319 (14)	−0.08179 (14)	0.92110 (10)	0.0262 (3)	
C2	0.20485 (15)	0.01702 (14)	0.85462 (10)	0.0267 (3)	
C3	0.12122 (16)	0.10425 (15)	0.88122 (11)	0.0303 (3)	
C4	0.14753 (18)	0.09740 (17)	0.97925 (12)	0.0368 (3)	
H4	0.0904	0.1559	0.9989	0.044*	
C5	0.25901 (18)	0.00335 (16)	1.04785 (11)	0.0360 (3)	
H5	0.2796	−0.0005	1.1155	0.043*	
C6	0.34170 (16)	−0.08577 (15)	1.01982 (10)	0.0304 (3)	
H6	0.4176	−0.1493	1.0682	0.036*	
C7	0.01064 (17)	0.19009 (17)	0.78777 (12)	0.0377 (4)	
H7A	0.0101	0.2934	0.8043	0.045*	
H7B	−0.0949	0.1513	0.7562	0.045*	
C8	0.07678 (17)	0.16885 (15)	0.71767 (11)	0.0346 (3)	
C9	−0.0406 (2)	0.1410 (2)	0.60728 (12)	0.0498 (4)	
H9A	0.0119	0.1132	0.5713	0.075*	
H9B	−0.1006	0.2290	0.5767	0.075*	
H9C	−0.1091	0.0628	0.6022	0.075*	
C10	0.1872 (2)	0.28869 (18)	0.73154 (13)	0.0449 (4)	
H10A	0.2604	0.3030	0.8041	0.067*	
H10B	0.1299	0.3783	0.7021	0.067*	
H10C	0.2427	0.2624	0.6973	0.067*	
C11	0.48288 (15)	−0.27410 (15)	0.94317 (10)	0.0288 (3)	
H11A	0.4348	−0.3362	0.9716	0.035*	
H11B	0.5754	−0.2287	1.0002	0.035*	
C12	0.52628 (16)	−0.36346 (15)	0.87960 (10)	0.0292 (3)	
O1	0.16890 (11)	0.03407 (10)	0.75592 (7)	0.0308 (2)	
O2	0.37837 (11)	−0.16642 (10)	0.87999 (7)	0.0300 (2)	
O3	0.48249 (14)	−0.34112 (12)	0.79128 (8)	0.0443 (3)	
O4	0.61833 (13)	−0.46912 (11)	0.93349 (8)	0.0387 (3)	
H4A	0.6434	−0.5164	0.8976	0.058*	
O5W	0.28781 (13)	−0.12943 (12)	0.65785 (8)	0.0354 (3)	
H5A	0.259 (2)	−0.079 (2)	0.6924 (15)	0.053*	
H5B	0.349 (2)	−0.191 (2)	0.7004 (15)	0.053*	

### Atomic displacement parameters (Å^2^)

**Table:**

	*U*^11^	*U*^22^	*U*^33^	*U*^12^	*U*^13^	*U*^23^
C1	0.0261 (6)	0.0248 (6)	0.0279 (6)	−0.0024 (5)	0.0140 (5)	−0.0013 (5)
C2	0.0286 (7)	0.0257 (7)	0.0248 (6)	−0.0032 (5)	0.0131 (5)	−0.0004 (5)
C3	0.0302 (7)	0.0274 (7)	0.0351 (7)	0.0001 (5)	0.0181 (6)	0.0001 (6)
C4	0.0434 (8)	0.0355 (8)	0.0406 (8)	0.0006 (7)	0.0282 (7)	−0.0035 (6)
C5	0.0464 (9)	0.0375 (8)	0.0300 (7)	−0.0025 (6)	0.0238 (7)	−0.0011 (6)
C6	0.0336 (7)	0.0292 (7)	0.0271 (7)	−0.0013 (6)	0.0148 (6)	0.0021 (6)
C7	0.0374 (8)	0.0356 (8)	0.0392 (8)	0.0077 (6)	0.0190 (7)	0.0032 (6)
C8	0.0383 (8)	0.0304 (7)	0.0315 (7)	0.0115 (6)	0.0153 (6)	0.0067 (6)
C9	0.0494 (10)	0.0519 (10)	0.0332 (8)	0.0185 (8)	0.0106 (7)	0.0038 (7)
C10	0.0559 (10)	0.0360 (8)	0.0472 (9)	0.0062 (7)	0.0297 (8)	0.0091 (7)
C11	0.0310 (7)	0.0279 (7)	0.0256 (6)	0.0040 (5)	0.0132 (5)	0.0053 (5)
C12	0.0307 (7)	0.0263 (7)	0.0295 (7)	0.0009 (5)	0.0147 (6)	0.0032 (6)
O1	0.0356 (5)	0.0299 (5)	0.0253 (5)	0.0089 (4)	0.0147 (4)	0.0050 (4)
O2	0.0343 (5)	0.0298 (5)	0.0247 (5)	0.0078 (4)	0.0145 (4)	0.0043 (4)
O3	0.0615 (7)	0.0419 (6)	0.0295 (5)	0.0174 (5)	0.0234 (5)	0.0061 (5)
O4	0.0495 (6)	0.0363 (6)	0.0347 (6)	0.0162 (5)	0.0249 (5)	0.0089 (5)
O5W	0.0475 (6)	0.0314 (6)	0.0309 (5)	0.0032 (5)	0.0227 (5)	0.0026 (4)

### Geometric parameters (Å, °)

**Table:**

C1—O2	1.3720 (16)	C8—C10	1.513 (2)
C1—C6	1.3903 (19)	C9—H9A	0.9800
C1—C2	1.3906 (18)	C9—H9B	0.9800
C2—O1	1.3738 (16)	C9—H9C	0.9800
C2—C3	1.3781 (19)	C10—H10A	0.9800
C3—C4	1.389 (2)	C10—H10B	0.9800
C3—C7	1.514 (2)	C10—H10C	0.9800
C4—C5	1.386 (2)	C11—O2	1.4147 (16)
C4—H4	0.9500	C11—C12	1.509 (2)
C5—C6	1.395 (2)	C11—H11A	0.9900
C5—H5	0.9500	C11—H11B	0.9900
C6—H6	0.9500	C12—O3	1.2082 (17)
C7—C8	1.548 (2)	C12—O4	1.3111 (16)
C7—H7A	0.9900	O4—H4A	0.8400
C7—H7B	0.9900	O5W—H5A	0.86 (2)
C8—O1	1.4868 (16)	O5W—H5B	0.85 (2)
C8—C9	1.511 (2)		
			
O2—C1—C6	127.47 (12)	C9—C8—C7	115.32 (13)
O2—C1—C2	115.14 (11)	C10—C8—C7	111.45 (13)
C6—C1—C2	117.38 (12)	C8—C9—H9A	109.5
O1—C2—C3	113.96 (12)	C8—C9—H9B	109.5
O1—C2—C1	123.09 (12)	H9A—C9—H9B	109.5
C3—C2—C1	122.93 (13)	C8—C9—H9C	109.5
C2—C3—C4	119.42 (13)	H9A—C9—H9C	109.5
C2—C3—C7	107.23 (12)	H9B—C9—H9C	109.5
C4—C3—C7	133.33 (13)	C8—C10—H10A	109.5
C5—C4—C3	118.58 (13)	C8—C10—H10B	109.5
C5—C4—H4	120.7	H10A—C10—H10B	109.5
C3—C4—H4	120.7	C8—C10—H10C	109.5
C4—C5—C6	121.54 (13)	H10A—C10—H10C	109.5
C4—C5—H5	119.2	H10B—C10—H10C	109.5
C6—C5—H5	119.2	O2—C11—C12	107.97 (11)
C1—C6—C5	120.07 (13)	O2—C11—H11A	110.1
C1—C6—H6	120.0	C12—C11—H11A	110.1
C5—C6—H6	120.0	O2—C11—H11B	110.1
C3—C7—C8	102.50 (11)	C12—C11—H11B	110.1
C3—C7—H7A	111.3	H11A—C11—H11B	108.4
C8—C7—H7A	111.3	O3—C12—O4	124.54 (13)
C3—C7—H7B	111.3	O3—C12—C11	124.88 (13)
C8—C7—H7B	111.3	O4—C12—C11	110.58 (11)
H7A—C7—H7B	109.2	C2—O1—C8	106.73 (10)
O1—C8—C9	105.86 (12)	C1—O2—C11	117.15 (10)
O1—C8—C10	106.75 (12)	C12—O4—H4A	109.5
C9—C8—C10	112.51 (14)	H5A—O5W—H5B	103.8 (18)
O1—C8—C7	104.03 (11)		
			
O2—C1—C2—O1	−2.53 (19)	C4—C3—C7—C8	166.32 (16)
C6—C1—C2—O1	178.40 (12)	C3—C7—C8—O1	22.35 (15)
O2—C1—C2—C3	175.87 (12)	C3—C7—C8—C9	137.81 (14)
C6—C1—C2—C3	−3.2 (2)	C3—C7—C8—C10	−92.32 (14)
O1—C2—C3—C4	−179.74 (12)	O2—C11—C12—O3	2.6 (2)
C1—C2—C3—C4	1.7 (2)	O2—C11—C12—O4	−177.34 (11)
O1—C2—C3—C7	1.47 (16)	C3—C2—O1—C8	13.73 (15)
C1—C2—C3—C7	−177.06 (13)	C1—C2—O1—C8	−167.74 (12)
C2—C3—C4—C5	0.7 (2)	C9—C8—O1—C2	−144.32 (13)
C7—C3—C4—C5	179.06 (15)	C10—C8—O1—C2	95.60 (13)
C3—C4—C5—C6	−1.5 (2)	C7—C8—O1—C2	−22.37 (14)
O2—C1—C6—C5	−176.62 (13)	C6—C1—O2—C11	2.41 (19)
C2—C1—C6—C5	2.3 (2)	C2—C1—O2—C11	−176.54 (11)
C4—C5—C6—C1	−0.1 (2)	C12—C11—O2—C1	173.93 (11)
C2—C3—C7—C8	−15.13 (15)		

### Hydrogen-bond geometry (Å, °)

**Table:**

*D*—H···*A*	*D*—H	H···*A*	*D*···*A*	*D*—H···*A*
O5W—H5A···O1	0.86 (2)	1.95 (2)	2.8104 (15)	173.0 (19)
O5W—H5B···O3	0.85 (2)	1.94 (2)	2.7888 (15)	176.5 (19)
O4—H4A···O5W^i^	0.84	1.71	2.5416 (15)	171

Symmetry codes: (i) −*x*+1, *y*−1/2, −*z*+3/2.
